# Supplementary material for: TREM2 expression level is critical for microglial state, metabolic capacity and efficacy of TREM2 agonism
Source: Nat Commun. 2026 Jan 24;17:2002. doi: 10.1038/s41467-026-68706-8 (PMC12936096; doi:10.1038/s41467-026-68706-8)
Supplement: Supplementary file 1 — Supplementary Information [file 41467_2026_68706_MOESM1_ESM.pdf]

SUPPLEMENTARY INFORMATION
FOR

TREM2 expression level is critical for microglial state, metabolic capacity and efficacy of TREM2 agonism

Astrid F Feiten[1,2,#], Kilian Dahm[3,4,5,#], Kai Schlepckow[2,#], Bettina van Lengerich[6], Jung H Suh[6], Anika Reifschneider[1], Benedikt Wefers[2], Laura M Bartos[7], Karin Wind-Mark[7], Lis de Weerd[2], Thomas Ulas[3,5,8], Elena De-Domenico[3,5,8], Pia Grundschöttel[3,5,8], Stefan Paulusch[3,5,8], Benjamin Tast[9], Tamisa Honda[9], Stephan A Müller[2,10], Matthias Becker[3,11], Igor Khalin[12,13], Alessio Ricci[12], Arthur Liesz[12,14], Bettina Brunner[2], Claudia Krenner[1], Katrin Buschmann[1], Brigitte Nuscher[1], Lena Spieth[2,15], Niklas Junker[2,15], Stefan A Berghoff[2,15], Sonnet S. Davis[6], Jonas J Neher[1,2,14], Wolfgang Wurst[2,16,17], Nikolaus Plesnila[12,14], Joseph W Lewcock[6], Mikael Simons[2,12,14,15], Stefan F Lichtenthaler[2,10,14], Gilbert Di Paolo[6], Matthias Brendel[2,7,14], Anja Capell[1,2], Kathryn M Monroe[6,*,+], Joachim L Schultze[3,5,8,*,+] and Christian Haass[1,2,14,*,+]

Contents:

Supplementary Figures 1-5

Supplementary Table 1

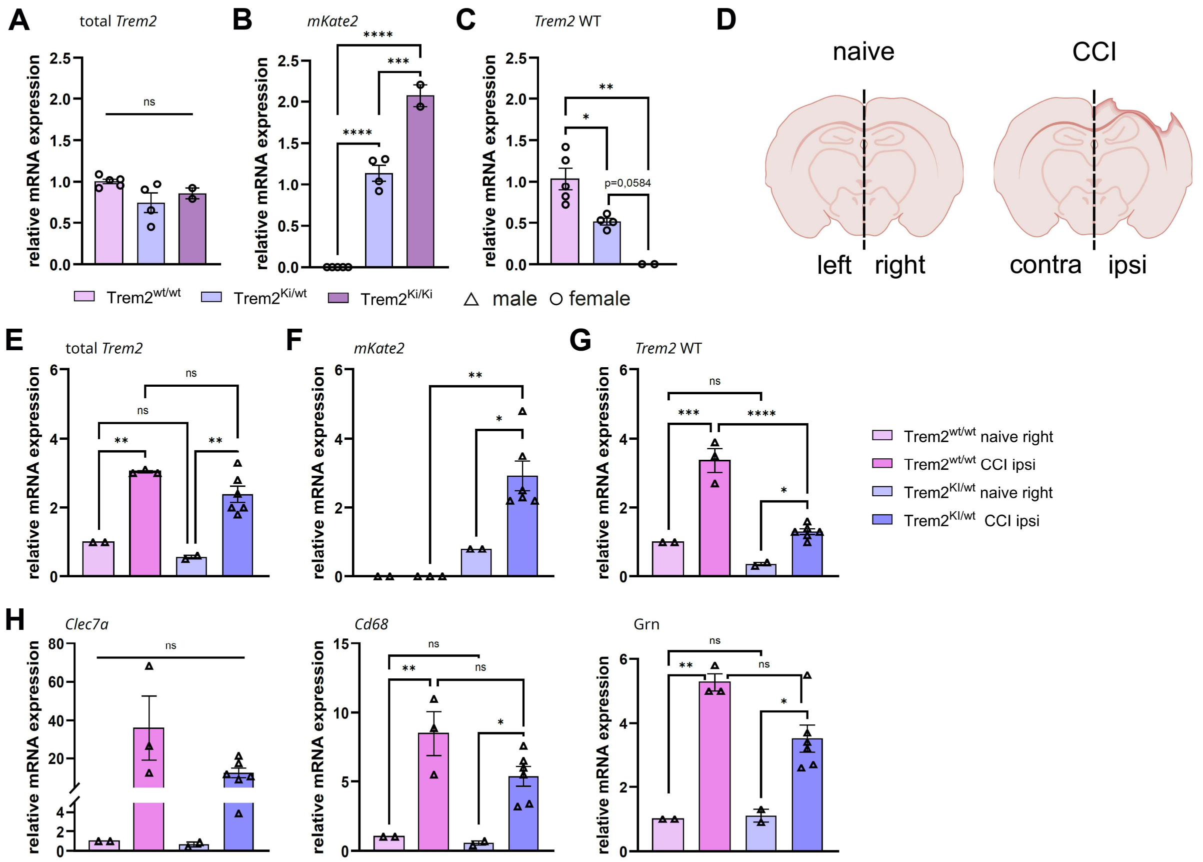

Supplementary Figure 1: **Expression of Trem2 and mKate2 reporter under basal conditions and upon controlled cortical impact.**

(A) – (C) mRNA expression levels for total *Trem2*, *mKate2* and the *Trem2* wildtype allele relative to either *Trem2*$^{\mathrm{wt/wt}}$ (A,C) or *Trem2*$^{\mathrm{Ki/wt}}$ (B) in *Trem2-mKate2* mice. N=5, N=4, and N=2 for *Trem2*$^{\mathrm{wt/wt}}$, *Trem2*$^{\mathrm{Ki/wt}}$, and *Trem2*$^{\mathrm{Ki/Ki}}$ mice, respectively. (D) Schematic representation of experimental groups of traumatic brain injury following controlled cortical impact. Created in BioRender. Mühlhofer, M. (2025) https://BioRender.com/mls61se. (E) – (H) mRNA expression levels for total *Trem2, mKate2*, *Trem2* wildtype allele, *Clec7a, Cd68* and *Grn* relative to *Trem2*$^{\mathrm{wt/wt}}$ (naïve, right) in *Trem2-mKate2* mice following controlled cortical impact. N=2, N=3, N=2, and N=6 for *Trem2*$^{\mathrm{wt/wt}}$ naïve right, *Trem2*$^{\mathrm{wt/wt}}$ CCI ipsi, *Trem2*$^{\mathrm{Ki/wt}}$ naïve right, and *Trem2*$^{\mathrm{Ki/wt}}$ CCI ipsi, respectively. *Hprt* was used as an endogenous expression control. Shown is the mean ± SEM. Statistical analyses were carried out by one-way ANOVA with Tukey's multiple comparisons test. Male and female mice are indicated by triangles and circles. Source data are provided as a Source Data file.

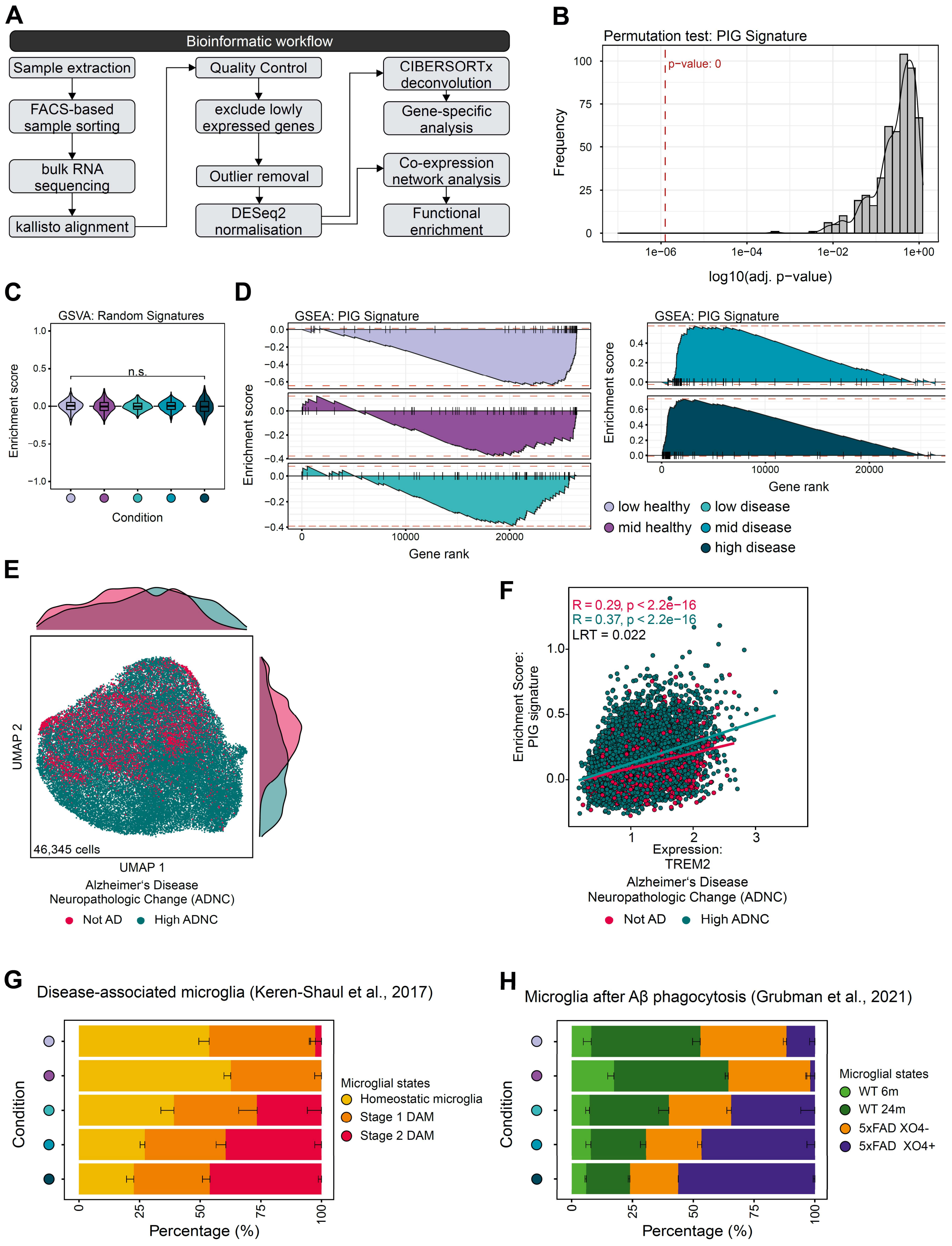

Supplementary Figure 2: Compositional shifts coincide with upregulation of microglial plaque-induced signature.

(A) Workflow of the bioinformatics analysis. (B) P-value distribution of gene set variation analysis (GSVA) enrichment results of 500 random, unique gene sets over all conditions on a log10 scale. Gene set size was based

on the size of the PIG signature. P-values were computed with a two-sided ANOVA followed by a Benjamini-Hochberg adjustment. The red dashed line represents the adjusted p-value from GSVA enrichment of the PIG signature. (C) Violin plot of GSVA enrichment scores of 500 random signatures coloured by condition. Boxplots show the 25%, 50% (median) and 75% percentile; whiskers denote 1.5 times the interquartile range (applies to all following boxplots). Statistics were computed by an unpaired, two-sided Wilcoxon test followed by a Benjamini-Hochberg adjustment and a summary of all comparisons is displayed. (D) Gene set enrichment analysis (GSEA) of the PIG signature coloured by condition. Ranking of genes is based on expression level statistics and the running sum is visualized. (E) Uniform Manifold Approximation and Projection (UMAP) of 46,345 human postmortem microglia of non-Alzheimer's disease (AD) donors and donors with high AD neuropathologic change (ADNC) from Gabitto et al.[47] (F) Linear regression analysis of the module enrichment score of the PIG signature against the TREM2 expression in TREM2-expressing human postmortem microglia. Dots and trend lines are coloured based on the ADNC. Pearson's correlation coefficients and p-values are documented for each trend line and the p-value of a likelihood ratio test (LRT) comparing a null linear mixed-effect model to a model split by the ADNC is indicated. (G) Stacked bar plot of microglial composition based on cell state deconvolution of the dataset by Keren-Shaul et al.[8] summarized per condition and coloured by activation state. Error bars represent the standard deviation. (H) Stacked bar plot of microglial composition based on cell state deconvolution of the dataset by Grubman et al.[48] summarized per condition and coloured by activation state. Error bars represent the standard deviation. Source data are provided as a Source Data file.

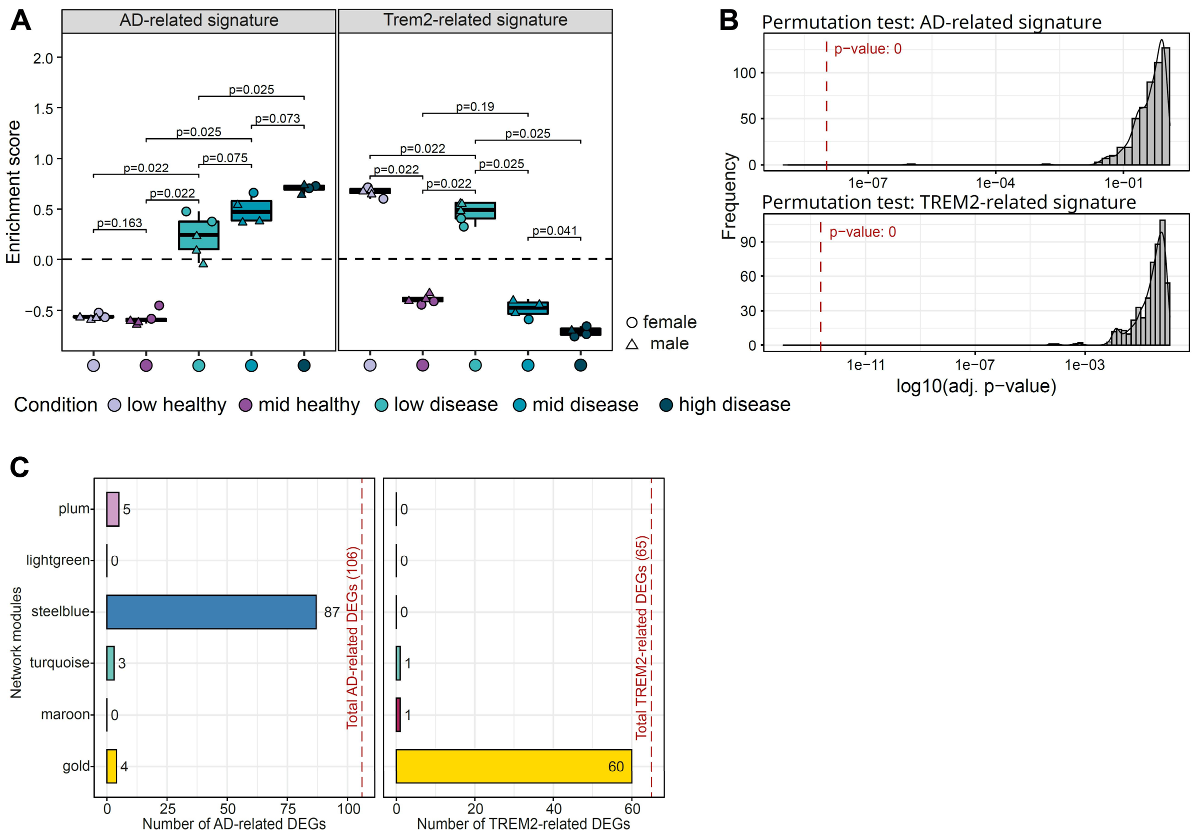

Supplementary Figure 3: Assessment of AD- and Trem2-related signatures.

(A) Boxplot of the gene set variation analysis (GSVA) enrichment scores of AD-related and TREM2-related DEGs coloured by condition. N = 5 mice for low healthy, mid healthy, and low disease conditions; N = 4 mice for mid disease and high disease conditions; age = 9 months for all conditions. Boxplots show the 25%, 50% (median) and 75% percentile, whiskers denote 1.5 times the interquartile range. Male and female mice are indicated by triangles and circles. Statistics were calculated with an unpaired, two-sided Wilcoxon test following a Benjamini-Hochberg adjustment. (B) P-value distribution of GSVA enrichment results of 500 random, unique gene sets over all conditions on a log10 scale. Gene set size was based on the size of the AD-related and TREM2-related signatures, respectively. P-values were computed with an ANOVA followed by a Benjamini-Hochberg adjustment. The red dashed lines represent the adjusted p-value from GSVA enrichment of the AD-related and TREM2-related signatures, respectively. (C) Barplot depicting the number of AD-related and TREM2-related DEGs included in each gene network module, respectively. The vertical lines represent the total numbers of the AD-related and TREM2-related DEGs. Source data are provided as a Source Data file.

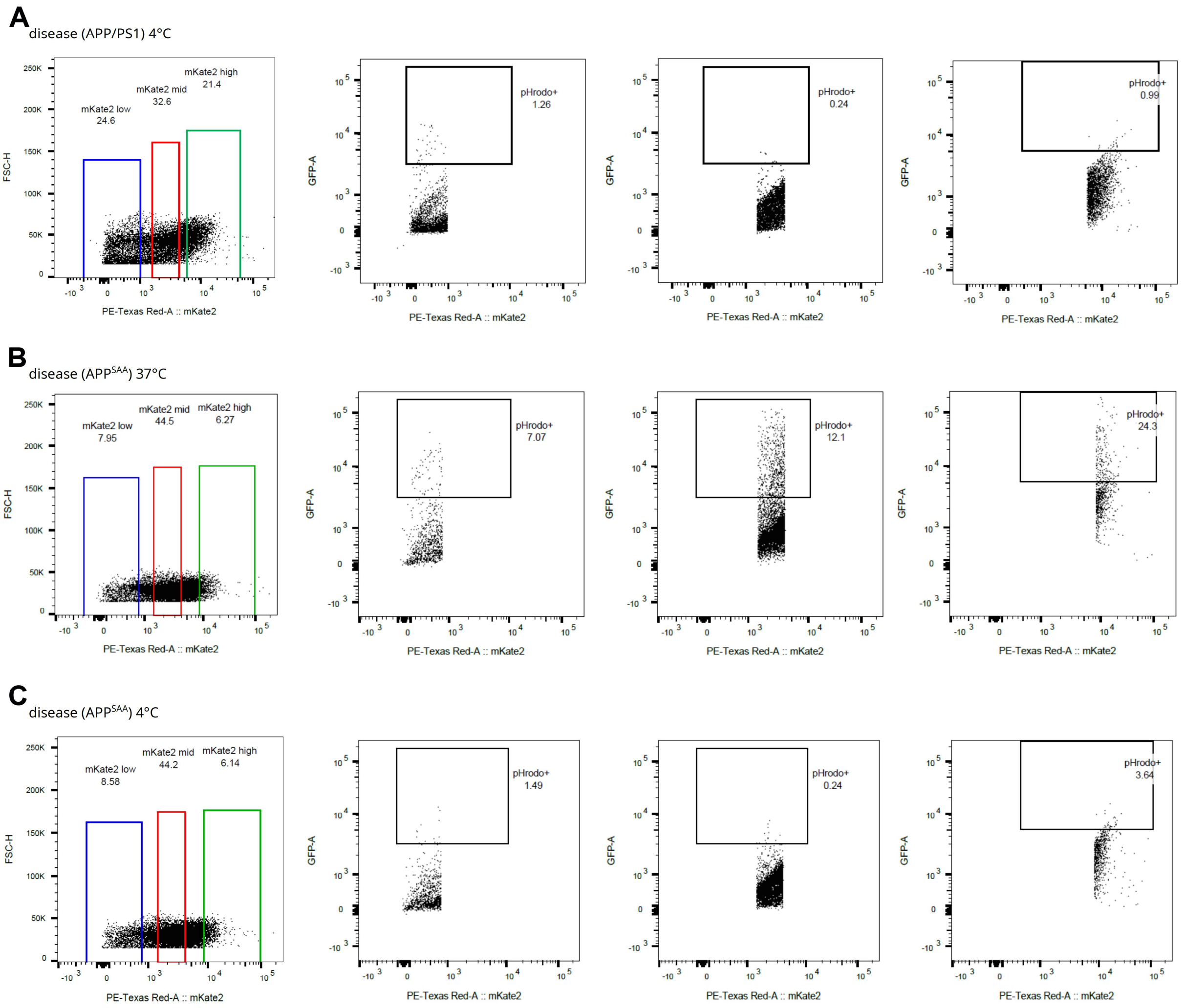

Supplementary Figure 4: Phagocytosis of pHrodo-myelin by microglial subpopulations in *APP/PS1* and *APP*$^{SAA/SAA}$ mice.

(A) FACS analysis of a 9.5-months-old *Trem2-mKate2*$^{KI/wt}$.*APP/PS1*$^{tg/wt}$ mouse showing that phagocytosis of pHrodo-labelled myelin is completely abolished at 4°C. The data were generated using isolated microglia from the same mouse as shown in Fig. 6A-C. (B) FACS data showing mKate2 low, mKate2 mid and mKate2 high gates in the CD11b-positive population as well as pHrodo gates in the mKate2 low, mid, and high subpopulations of a 13-months old *Trem2-mKate2*$^{KI/wt}$.*APP*$^{SAA/SAA}$.*hTfR*$^{KI/KI}$ mouse from which percentages of pHrodo-positive cells were quantified. (C) FACS analysis of a 13-months-old *Trem2-mKate2*$^{KI/wt}$.*APP*$^{SAA/SAA}$.*hTfR*$^{KI/KI}$ mouse showing that phagocytosis of pHrodo-labelled myelin is completely abolished at 4°C. The data were generated using isolated microglia from the same mouse as shown in Supplementary Fig.4B.

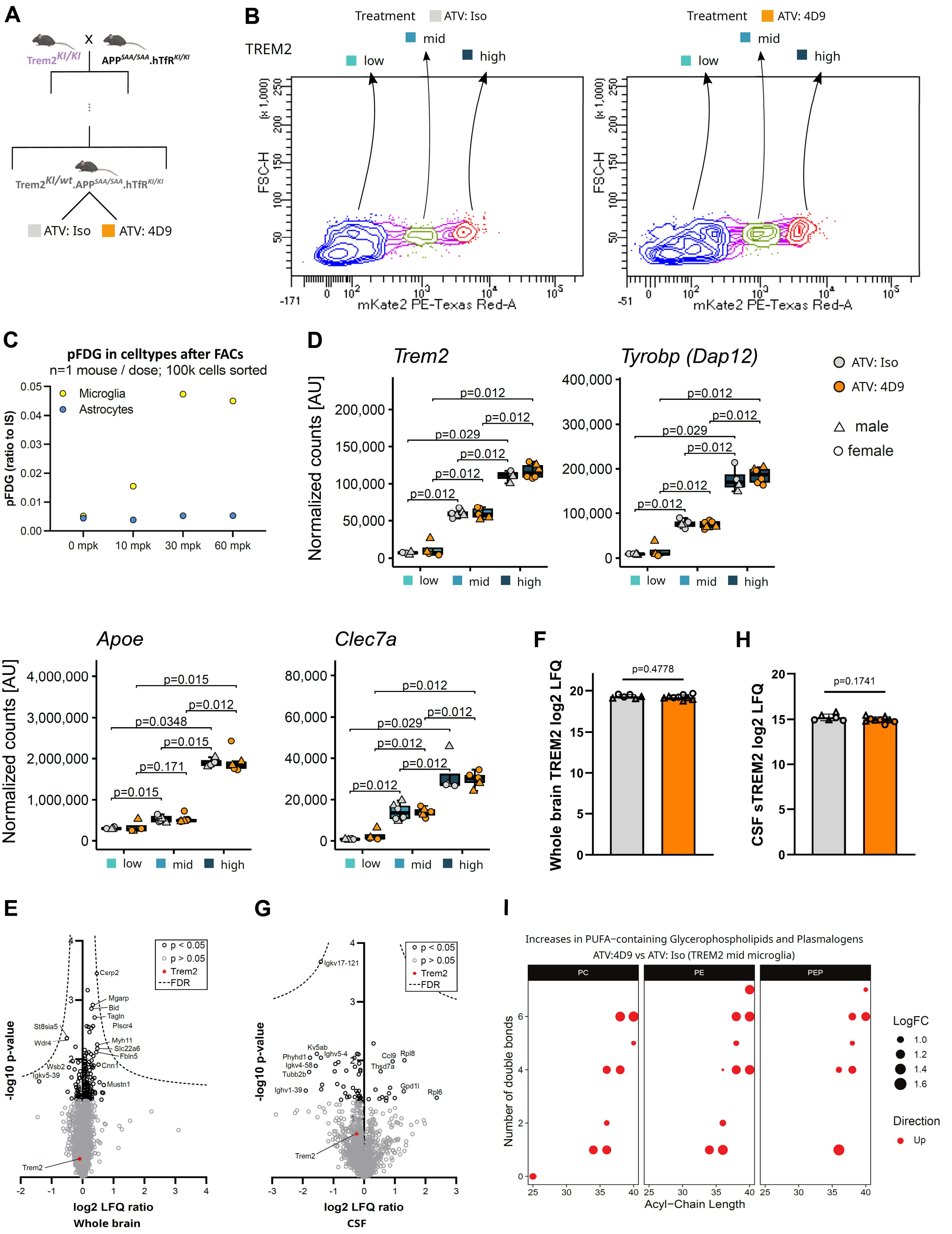

Supplementary Figure 5: Transcriptomic and proteomic analyses of *APP*$^{SAA/SAA}$ mice upon chronic TREM2 antibody treatment.

(A) *Trem2-mKate2*$^{KI/wt}$.*APP*$^{SAA/SAA}$.*hTfR*$^{KI/KI}$ mice were treated with either ATV:isotype (grey) or ATV:4D9 (orange) at 1 mg/kg; n = 6 mice per treatment group. (B) Example contour FACS plots which were used to define

and sort microglia into low mKate2, mid mKate2, and high mKate2 subpopulations. (C) Example graph of the level of pFDG that could be measured after dosing with different concentrations of FDG in microglia or astrocytes respectively. This graph is derived from method establishment with wildtype animals without antibody treatment. (D) Boxplot of normalized gene expression of *Trem2*, *Tyrobp* (transcript for Dap12), *ApoE*, and *Clec7a* per stratified microglia subpopulation and split and coloured by treatment. Statistics were computed using an unpaired, two-sided Wilcoxon test followed by a Benjamini-Hochberg adjustment. For the RNA-seq data of *Trem2-mKate2*$^{KI/wt}$.*APP*$^{\mathrm{SAA/SAA}}$.*hTfR*$^{KI/KI}$ mice: N=4 for low and high mKate2 with ATV:Isotype treatment and low mKate2 with ATV:4D9 treatment; N=6 for mid mKate2 with ATV:Isotype treatment and mid and high mKate2 with ATV:4D9 treatment; age = 12 months. (E) Volcano Plot for whole brain RIPA extracts comparing ATV:4D9 and ATV:isotype treatments. The minus log10 transformed p-values of each protein are plotted against the log2 transformed label free quantification (LFQ) ratios for each protein. Proteins with a p-value less than 0.05 are indicated as black circles, whereas those with a p-value higher than 0.05 are displayed as grey circles. The dashed hyperbolic curves are the thresholds of the permutation based false discovery rate correction for multiple hypotheses (p = 0.05; s0 =0.1). Selected proteins are labeled with their UniProt gene names. (F) TREM2 quantification in whole brain lysates by mass spectrometry. Shown is the mean ± SD. Statistical analysis was carried out by an unpaired two-tailed t test. (G) Volcano Plot for CSF samples comparing ATV:4D9 and ATV:isotype control treatments. (H) sTREM2 quantification in CSF by mass spectrometry. Shown is the mean ± SD. Statistical analysis was carried out by an unpaired two-tailed t test. (I) Log fold changes in PUFA-containing phospholipids and plasmalogens by ATV:4D9 vs ATV:isotype in mid-TREM2 microglia. Male and female mice are indicated by triangles and circles in (D,F,H). Source data are provided as a Source Data file.

Antibody	Company	Catalog number	Species	Dilution	Application
mKate2	Evrogen	AB233	Rabbit	1:500	Histology
Trem2	R&D Systems	AF1729	Sheep	1:200	Histology
Iba-1	Novusbio	NB100-1028	Goat	1:300	Histology
Aβ 1-40	Cell Signaling Technologies	NAB288	Mouse	1:1,000	Histology
Alexa647	Invitrogen	A32795	Rabbit	1:500	Histology
Alexa555	Invitrogen	A32773	Mouse	1:500	Histology
Alexa488	Invitrogen	A32814	Goat	1:500	Histology
Alexa488	Invitrogen	A11015	Sheep	1:500	Histology
Trem2	Cell Signaling Technologies	76765	Rabbit	1:1,000	Immunoblot
mKate2	OriGene	TA150072	Rabbit	1:4,000	Immunoblot
Actin	Sigma	A5316	Mouse	1:20,000	Immunoblot
HRP-conjugated anti-rabbit 2nd	Promega	W401B		1:10,000	Immunoblot
HRP-conjugated anti-mouse 2nd	Promega	W402B		1:10,000	Immunoblot
CD11b-BV421	Biolegend	101251	Rat	1:100	FACS
CD45-APC	BD Pharmingen	559864	Rat	1:100	FACS
CD16/32	Biolegend	101320	Rat	1:100	FACS

Supplementary Table 1: Summary of antibodies used for histology, immunoblotting and FACS.
