# Supplementary material for: TREM2 expression level is critical for microglial state, metabolic capacity and efficacy of TREM2 agonism
Source: Nat Commun. 2026 Jan 24;17:2002. doi: 10.1038/s41467-026-68706-8 (PMC12936096; doi:10.1038/s41467-026-68706-8)
Supplement: Supplementary file 2 — Description of Additional Supplementary Files [file 41467_2026_68706_MOESM2_ESM.pdf]

Description of Additional Supplementary Files

File name: Supplementary Data 1

Description: AD-related DEGs and Trem2-related DEGs.

File name: Supplementary Data 2

Description: TREM2 expression level dependent differences in metabolite abundances in healthy and disease mice.

File name: Supplementary Data 3

Description: Differences in metabolite abundances in microglial subpopulations upon chronic TREM2 antibody treatment.
