# Supplementary material for: TREM2 expression level is critical for microglial state, metabolic capacity and efficacy of TREM2 agonism
Source: Nat Commun. 2026 Jan 24;17:2002. doi: 10.1038/s41467-026-68706-8 (PMC12936096; doi:10.1038/s41467-026-68706-8)
Supplement: Supplementary file 6 — Reporting Summary [file 41467_2026_68706_MOESM6_ESM.pdf]

nature portfolio

Corresponding author(s): Kathryn M Monroe, Joachim L Schultze, Christian Haass

Last updated by author(s): Dec 10, 2025

Reporting Summary

Nature Portfolio wishes to improve the reproducibility of the work that we publish. This form provides structure for consistency and transparency in reporting. For further information on Nature Portfolio policies, see our Editorial Policies and the Editorial Policy Checklist.

Statistics

For all statistical analyses, confirm that the following items are present in the figure legend, table legend, main text, or Methods section.

n/a	Confirmed	
☐	☒	The exact sample size (*n*) for each experimental group/condition, given as a discrete number and unit of measurement
☐	☒	A statement on whether measurements were taken from distinct samples or whether the same sample was measured repeatedly
☐	☒	The statistical test(s) used AND whether they are one- or two-sided<br>*Only common tests should be described solely by name; describe more complex techniques in the Methods section.*
☐	☒	A description of all covariates tested
☐	☒	A description of any assumptions or corrections, such as tests of normality and adjustment for multiple comparisons
☐	☒	A full description of the statistical parameters including central tendency (e.g. means) or other basic estimates (e.g. regression coefficient) AND variation (e.g. standard deviation) or associated estimates of uncertainty (e.g. confidence intervals)
☐	☒	For null hypothesis testing, the test statistic (e.g. *F*, *t*, *r*) with confidence intervals, effect sizes, degrees of freedom and *P* value noted<br>*Give P values as exact values whenever suitable.*
☒	☐	For Bayesian analysis, information on the choice of priors and Markov chain Monte Carlo settings
☒	☐	For hierarchical and complex designs, identification of the appropriate level for tests and full reporting of outcomes
☐	☒	Estimates of effect sizes (e.g. Cohen's *d*, Pearson's *r*), indicating how they were calculated

Our web collection on statistics for biologists contains articles on many of the points above.

Software and code

Policy information about availability of computer code

Data collection

No software was used for data collection.

Data analysis

Transcriptomics (APP-PS1):
bcl2fastq2 (v2.20), kallisto (v0.44.2), R (v4.1.0 bulkRNA-seq; v4.2.0 hcocena), RStudio (1.4.1717 bulkRNA-seq; 2022.02.3+492 hcocena), tximport (v1.20.0), DESeq2 (v1.32.0), IHW (v1.20.0), apeglm (v1.14.0), CIBERSORTx (https://cibersortx.stanford.edu/), GSVA (v1.40.1), fgsea (v1.18.0), ggplot2 (v3.3.5 bulkRNA-seq; v3.3.6 hcocena), ggpubr (v0.4.0), hcocena (v1.0.0), pheatmap (v1.0.12), rstatix (v0.7.0), UpSetR (v1.4.0), Seurat (v4.0.4), lmtest (v0.9-38), lm4 (v1.1-27.1), dplyr (v.1.0.7)

Transcriptomics (APP-SAA):
bcl2fastq2 (v2.20), kallisto (v0.48.0), R (v4.3.0), R Studio (1.4.1717), tximport (v1.28.0), DESeq2 (v1.40.2), rstatix (v0.7.2)

All computational analyses of the RNA-seq data were performed using R programming languages.
All original code to reproduce key steps of the RNA-seq analysis has been deposited at GitLab under https://gitlab.dzne.de/ag-ulas/Trem2-reporter-mouse-model and Zenodo under https://doi.org/10.5281/zenodo.17793705.

Lipidomics/Metabolomics:
MultiQuant 3.02 (Sciex)
R Studio 2024.04.0+735

Proteomics:

Thermo Xcalibur 4.5.474.0, Bruker Hystar 6.2.1.13, DIA-NN 2.0.2, Perseus 1.6.2.3, Microsoft Excel 2021

For manuscripts utilizing custom algorithms or software that are central to the research but not yet described in published literature, software must be made available to editors and reviewers. We strongly encourage code deposition in a community repository (e.g. GitHub). See the Nature Portfolio guidelines for submitting code & software for further information.

Data

Policy information about availability of data

All manuscripts must include a data availability statement. This statement should provide the following information, where applicable:

- Accession codes, unique identifiers, or web links for publicly available datasets
- A description of any restrictions on data availability
- For clinical datasets or third party data, please ensure that the statement adheres to our policy

All data analysed and interpreted in this study are included in this published article or its supplementary files. The RNA-seq data generated in this study have been deposited under GEO accession codes GSE271074 (APP/PS1 mice) [https://www.ncbi.nlm.nih.gov/geo/query/acc.cgi?acc=GSE271074] and GSE295016 (AppSAA mice) [https://www.ncbi.nlm.nih.gov/geo/query/acc.cgi?acc=GSE295016]. The proteomic data generated in this study have been deposited to the ProteomeXchange Consortium via the PRIDE partner repository under accession code PXD065438 [https://www.ebi.ac.uk/pride/archive/projects/PXD065438]. The LC/MS data generated in this study have been deposited in the Metabolomics Workbench database under accession codes ST003501 (APP/PS1 mice) and ST004230 (AppSAA mice) [Project DOI: 10.21228/M83F9Q]. The sc/snRNA-seq data used in this study are available under GEO accession codes GSE98969 [https://www.ncbi.nlm.nih.gov/geo/query/acc.cgi?acc=GSE98969] and GSE165306 [https://www.ncbi.nlm.nih.gov/geo/query/acc.cgi?acc=GSE165306] and at CELLxGENE [(https://cellxgene.cziscience.com/collections/1ca90a2d-2943-483d-b678-b809bf464c30]. Source data are provided with this paper.

Research involving human participants, their data, or biological material

Policy information about studies with human participants or human data. See also policy information about sex, gender (identity/presentation), and sexual orientation and race, ethnicity and racism.

Reporting on sex and gender	This study did not involve human participants, their data, or biological material. Therefore, this information has not been collected.
Reporting on race, ethnicity, or other socially relevant groupings	This study did not involve human participants, their data, or biological material. Therefore, this information is not available.
Population characteristics	This study did not involve human participants, their data, or biological material. Therefore, this information is not available.
Recruitment	This study did not involve human participants, their data, or biological material. Therefore, this information is not available.
Ethics oversight	This study did not involve human participants, their data, or biological material. Therefore, this information is not available.

Note that full information on the approval of the study protocol must also be provided in the manuscript.

Field-specific reporting

Please select the one below that is the best fit for your research. If you are not sure, read the appropriate sections before making your selection.

☒ Life sciences ☐ Behavioural & social sciences ☐ Ecological, evolutionary & environmental sciences

For a reference copy of the document with all sections, see nature.com/documents/nr-reporting-summary-flat.pdf

Life sciences study design

All studies must disclose on these points even when the disclosure is negative.

Sample size	No sample-size calculation was performed. Mouse numbers were generally chosen such that statistically significant differences between exerimental groups could be obtained.
Data exclusions	No data were excluded from the analyses.
Replication	In all experiments, mouse numbers were chosen such that statistically significant differences between exerimental groups were obtained.
Randomization	In general, equal numbers of female and male mice were allocated into experimental groups.
Blinding	Investigators were not blinded to group allocation during data collection and analysis.

Reporting for specific materials, systems and methods

We require information from authors about some types of materials, experimental systems and methods used in many studies. Here, indicate whether each material, system or method listed is relevant to your study. If you are not sure if a list item applies to your research, read the appropriate section before selecting a response.

Materials & experimental systems

n/a	Involved in the study
☐	☒ Antibodies
☐	☒ Eukaryotic cell lines
☒	☐ Palaeontology and archaeology
☐	☒ Animals and other organisms
☒	☐ Clinical data
☒	☐ Dual use research of concern
☒	☐ Plants

Methods

n/a	Involved in the study
☒	☐ ChIP-seq
☐	☒ Flow cytometry
☒	☐ MRI-based neuroimaging

Antibodies

Antibodies used

Antibodies used in the study:
Anti-mKate2: Evrogen; species: rabbit; catalog number: AB233
Anti-Trem2: R&D Systems; species: sheep; catalog number: AF1729
Anti-Iba1: Novusbio; species: goat; catalog number: NB100-1028
Anti-Abeta(1-40): Cell Signaling Technologies; species: mouse; catalog number: 2450; clone name: NAB228
Alexa-647 secondary antibody: Invitrogen; species: rabbit; catalog number: A-21245
Alexa-555 secondary antibody: Invitrogen; species: mouse; catalog number: A-21424
Alexa-488 secondary antibody: Invitrogen; species: goat; catalog number: A-11055
Alexa-488 secondary antibody: Invitrogen; species: sheep; catalog number: A-11015
Anti-Trem2: Cell Signaling Technologies; species: rabbit; catalog number: 76765
Anti-Trem2: Invitrogen; species: rabbit; catalog number: MA5-31267
Anti-mKate2: OriGene; species: rabbit; catalog number: TA150072
Anti-Actin: Sigma; species: mouse; catalog number: A5316
HRP-conjugated anti-rabbit 2nd antibody: Promega; catalog number: W401B
HRP-conjugated anti-mouse 2nd antibody: Promega; catalog number: W402B
CD11b-BV421: Biolegend; species: rat; catalog number: 101251
CD45-APC: BD Pharmingen; species: rat; catalog number: 559864
CD16/32: Biolegend; species: rat; catalog number: 101320
Treatment antibodies ATV:ISO and ATV:4D9 pertaining to Figure 7 were provided by DENALI Therapeutics.

Validation

Rabbit anti-mKate2 (AB233) (cited on manufacturer's website for use in immunocytochemistry)
Sheep anti-Trem2 (validated in previous work from our laboratory: Parhizkar et al, 2019, Schlepckow et al, 2020; cited on manufacturer's website for immunofluorescence of amyloid mouse models; several citations for immunofluorescence on citeab.com)
Goat anti-Iba1 (Knockout validated and validated in various immunofluorescence applications as cited on manufacturer's website; several dozens of citations for immunofluorescence on citeab.com)
Mouse anti-Abeta(1-40) (several citations for immunofluorescence on citeab.com)
Rabbit anti-Trem2 (76765) (cited on manufacturer's website for immunoblotting; several citations for immunoblotting on citeab.com)
Rabbit anti-Trem2 (MA5-31267) (we validated this antibody by including a Trem2 KO sample as shown in the immunoblot in Supplementary Fig. 5G)
Rabbit anti-mKate2 (TA150072) (one citation for immunoblotting on citeab.com)
Mouse anti-Actin (cited on manufacturer's website for immunoblotting; several citations for immunoblotting on citeab.com)
Rat CD11b-BV421 (validated in previous work from our collaborators at DENALI Therapeutics: Van Lengerich et al, 2023; several citations for FACS on citeab.com)
Rat CD45-APC (several citations for FACS on citeab.com)
Rat CD16/32 (validated in previous work from our collaborators at DENALI Therapeutics: Van Lengerich et al, 2023; several citations for FACS on citeab.com)

Eukaryotic cell lines

Policy information about cell lines and Sex and Gender in Research

Cell line source(s)	HeLa cells were used to generate data as shown in Figure 1B-D.
Authentication	HeLa (CCL-2) ATCC, not additionally authenticated
Mycoplasma contamination	All cell lines are tested negative for mycoplasma contamination
Commonly misidentified lines (See ICLAC register)	non commonly misidentified cell line were used

Animals and other research organisms

Policy information about studies involving animals; ARRIVE guidelines recommended for reporting animal research, and Sex and Gender in Research

Laboratory animals	C57BL/6J mouse zygotes were used to generate Trem2-mKate2 knockin mice.<br><br>Further mouse strains used in this study (all on C57BL/6J background):<br>APPPS1 (wt/tg) x Trem2-mKate2 (wt/ki) mice: 4 months (Figures 1H,I); 9 months (Figures 2, 3, and 5); 14 months (Figure 4); 9.5 months (Figure 6).<br>APP-SAA (ki/ki) x hTfR (ki/ki) x Trem2-mKate2 (wt/ki) mice: 13 months (Figure 6); 8 months at the beginning of antibody dosing; 12 months at the time of sacrifice (Figure 7).
Wild animals	This study did not involve wild animals.
Reporting on sex	Sex was considered in the study design, i.e., equal numbers of male and female mice were employed whenever this was possible. With the mouse numbers employed we did not find any sex-specific differences.<br>Overall mouse numbers:<br>Figures 1H,I: 3 female and 3 male APPPS1 (wt/tg) x Trem2-mKate2 (wt/ki) mice.<br>Figures 2, 3, and 5 plus Supplementary Figure 3: 3 male and 2 female APPPS1 (wt/tg) x Trem2-mKate2 (wt/ki) mice plus 1 male and 6 female APPPS1 (wt/wt) x Trem2-mKate2 (wt/ki) mice.<br>Figure 4: 3 female and 3 male Trem2-mKate2 (wt/ki) mice; 4 female and 1 male APPPS1 (wt/tg) x Trem2-mKate2 (wt/ki) mice.<br>Figure 6 plus Supplementary Figure 4: 1 female and 5 male APPPS1 (wt/tg) x Trem2-mKate2 (wt/ki) mice; 2 female and 2 male APPSAA<br>(ki/ki) x hTfR (ki/ki) x Trem2-mKate2 (wt/ki) mice.<br>Figure 7 + Supplementary Figure 5D+I: 4 female and 2 male APP-SAA (ki/ki) x hTfR (ki/ki) x Trem2-mKate2 (wt/ki) mice dosed with ATV:4D9 plus 3 female and 3 male APP-SAA (ki/ki) x hTfR (ki/ki) x Trem2-mKate2 (wt/ki) mice dosed with ATV:ISO.<br>Supplementary Figure 1A-C: female mice only; Supplementary Figure 1E-H: male mice only.<br>Supplementary Figure 5E+F+H: 4 female and 5 male APP-SAA (ki/ki) x hTfR (ki/ki) x Trem2-mKate2 (wt/ki) mice dosed with ATV:4D9 plus 3 female and 3 male APP-SAA (ki/ki) x hTfR (ki/ki) x Trem2-mKate2 (wt/ki) mice dosed with ATV:ISO.
Field-collected samples	This study did not involve samples collected from the field.
Ethics oversight	All animal experiments were approved by the Ethical Review Board of the Government of Upper Bavaria (animal licenses ROB-55.2-2532.Vet_02-17-75, ROB-55.2-2532.Vet_02-22-125, ROB-55.2-2532.Vet_02-16-121, and ROB-55.2-2532.Vet_02-18-39).

Note that full information on the approval of the study protocol must also be provided in the manuscript.

Plants

Seed stocks	No plant specimens were collected from the field.
Novel plant genotypes	Novel plant genotypes were not produced in this study.
Authentication	This information is not available since no plant specimens were collected from the field and novel plant genotypes were not produced in this study.

Flow Cytometry

Plots

Confirm that:

☒ The axis labels state the marker and fluorochrome used (e.g. CD4-FITC).

☒ The axis scales are clearly visible. Include numbers along axes only for bottom left plot of group (a 'group' is an analysis of identical markers).

☐ All plots are contour plots with outliers or pseudocolor plots.

☐ A numerical value for number of cells or percentage (with statistics) is provided.

Methodology

Sample preparation	Microglia isolation was performed using the MACS system (Miltenyi Biotec). No CD11b enrichment step was performed as the intrinsic mKate2 reporter is only expressed in TREM2 expressing (microglia) cells. Hence, FACS was performed based on the mKate2 fluorescence signal as a proxy for the TREM2 expression of individual cells.Cells were sorted into low, mid and

high expressing subpopulations. The sorted fractions were spun down and the pellets stored at -80°C before being processed for further analysis.
Regarding ex vivo phagocytosis, cells were stained with CD11b-BV421, CD45-APC, and CD16/32 (Fc blocker) upon pHrodo-myelin uptake and subsequently analyzed.

Instrument	BDFACSAriaIII (APPPS1 (wt/tg) x Trem2-mKate2 (wt/ki) mice) and BDFACSAria Fusion (APP-SAA (ki/ki) x hTfR (ki/ki) x Trem2-mKate2 (wt/ki) mice).
Software	BD FACSDiva 8.0.1, FlowJo software (V10)
Cell population abundance	Purity assessment of the sorted samples was done by re-introducing the sorted cells to the instrument, 500-1000 of sorted cells were analysed under the same setup, gating strategy, voltage etc.
Gating strategy	Cell population gate is placed on FSC-A,SSC-A, then singlet cells are gated based on FSC-A,FSC-H plot to delete the doublets. DAPI (BV421-A),FSC-A plot is used to gate out the live cells. mKate2 signal is detected on PE-Texas Red channel and 3 distinct gates for low, med, high signal are defined according to intensity of signal. Unstained control was used to set the gate for live cells, negative/positive control is used for mKate2 positive cells.<br>For better visualization contour plot is included.<br>Regarding ex vivo phagocytosis, we followed this gating strategy: CD11b (BV-421)-> mKate2 (Texas Red)-> pHrodo (GFP). In this experiment, we did not include a viability dye.

☒ Tick this box to confirm that a figure exemplifying the gating strategy is provided in the Supplementary Information.
